# Diabetes in Algeria and challenges for health policy: a literature review of prevalence, cost, management and outcomes of diabetes and its complications

**DOI:** 10.1186/1744-8603-10-11

**Published:** 2014-02-24

**Authors:** Larbi Lamri, Erofile Gripiotis, Alessandra Ferrario

**Affiliations:** 1 University of Algiers, Rue Didouche Mourad, Algiers, Algeria; 2 London School of Economics and Political Science, Houghton Street, London WC2A 2AE, UK

**Keywords:** Diabetes, Costs, Algeria, Prevalence, Diabetes management

## Abstract

**Background:**

Diabetes has become an increasingly prevalent and severe public health issue in Algeria. This article investigates the prevalence, the cost and the management of this disease. Its first objective is to better understand the burden (both from an epidemiological and economic perspective) and management of diabetes. The second objective is to understand the health policy strategy adopted by Algeria in order to respond to the disease.

**Methods:**

We conducted a literature review of prevalence, costs, management and outcomes of diabetes and its complications. This was complemented by data compilations and results of expert consultations.

**Results:**

The epidemiology of diabetes is continually evolving and is becoming more problematic. The national evidence suggests that the prevalence of diabetes in Algeria has increased from 6.8% in 1990 to 12.29% in 2005, but is quite higher among certain groups and areas of the country. This disease affects all population groups, especially 35–70 year olds, who constitute a large segment of the working population. There are very few estimates of the cost of diabetes. These include a 1998 study on the total cost of type 1 diabetes (USD 11.6 million, which, inflated to 2013 value, totals to USD 16.6 million), a study on the cost of complications in 2010 (at 2013 value, ranging from USD 141 for first-year treatment of peripheral vascular disease to USD 30,441 for first-year cost of renal transplantation) and the 2013 IDF estimates of total cost of type 1 and type 2 diabetes (USD 513 million).

**Conclusions:**

As the prevalence of diabetes continues to increase, the financial burden will increasingly weigh heavily on social security resources and the government budget. Future priorities must focus on empowering general practitioners in treating type 2 diabetes, improving screening of diabetes and its complications, tackling the growing obesity epidemic, strengthening health information systems and implementing the national diabetes prevention and control plan.

## Background

Similar to much of the developing world, Algeria is currently undergoing an epidemiological transition. While mal- and undernutrition and infectious diseases used to be the main causes of poor health, today there is a higher proportion of chronic, non-communicable diseases (NCDs), including diabetes mellitus, cardiovascular disease, cancer and respiratory diseases. According to estimates for Algeria from the World Health Organization (WHO), NCDs accounted for 63% of all deaths in 2010 [1]. By comparison, a smaller proportion of overall mortality (29%) that year was attributed to communicable diseases, maternal, perinatal and nutritional conditions (injuries represented the remaining 8%) [1].

Type 2 diabetes, once considered a disease of industrialised nations, is now becoming increasingly prevalent in Algeria and other emerging countries, ranking as the fourth most prevalent non-communicable disease [2]. This upward trend in diabetes prevalence is contributing to a double burden of disease, which has significant implications not only for population health but also in socio-economic contexts.

Assessment of the prevalence, complications, costs and outcomes of diabetes in Algeria has been rather limited by insufficient data and projections on the significance of the disease from a public health perspective. A literature review on the prevalence of diabetes mellitus was published in 2008 [3]. However, evidence on the burden and management of type 1 and type 2 diabetes in Algeria is fragmentary. This study aims to address this gap by conducting a comprehensive literature review of available data sources on the burden and management of diabetes (including the prevalence of both type 1 and type 2, complications, costs, health outcomes and policies developed in response to the disease) and using this as a basis to formulate evidence-based policy recommendations for Algeria.

### Overview of Algerian health care system

Algeria is the largest country in Africa and is estimated to have a population of around 38 million inhabitants [4]. Approximately 70% of the population lives in the coastal region, north of the minority who live in the Sahara region mainly concentrated in oases, while 1.5 million belong to nomadic communities; almost 30% of Algerians are under 15 years of age [2].

The Algerian health system has both public and private health sectors. The public health sector is accessible and free-of-charge for all Algerian citizens; it is dually financed by government contributions and social insurance. There are 12.1 physicians and 19.5 nurses/midwives, respectively, per 10,000 population [5]. The number of health care facilities varies from one area of the country to another, depending on the size of the local population.

In 2011, Algeria’s total expenditure on health per capita was USD 354 at purchasing-power parity, which represented 3.9% of gross domestic product [6]. In that same year, general government and private expenditures on health represented 80.8% and 19.2% of total health expenditure, respectively [6]. Of general government expenditure on health, 32.4% was constituted of social security funds while 94.7% of private expenditure on health was out-of-pocket spending [6]. There is a national medical insurance scheme run by the Caisse Nationale des Assurances Sociales des Travailleurs Salariés (CNAS) that covers 90% of the entire population [7]. Public health insurance is available for salaried employees and independent workers such as traders.

Medicines are reimbursed at 80% of the reference price; medical procedures (consultations, examinations and tests) are also reimbursed at 80%. All patients who are employed in the formal sector and have chronic diseases (including diabetes) are reimbursed 100% of the costs of care and medicines (patients receive medicines free-of-charge as they are covered by health insurers which pay pharmacies a fixed price). Low-income formal sector workers (i.e. with health insurance) are identified by the Algerian Ministry of National Solidarity and Family and local authorities (districts and sub-districts). Individuals belonging to this patient group are provided a card which grants them access to free medication and care; the ministry and local authorities pay for their health care costs.

## Methods

This study is based primarily on a comprehensive literature review, internet research and consultations with relevant experts.

The literature review was conducted in November 2013 using English as well as French keywords. The following search engines were accessed: PubMed, Web of Science, Scopus, Google Scholar and Google.

English keyword searches in PubMed used the following keywords: Algeria OR Algerian AND diabetes OR diabetic OR neuropathy OR retinopathy OR nephropathy OR “renal replacement” OR “chronic kidney” OR “diabetic foot” OR “foot ulcer” AND prevalence OR expenditure OR expenses OR cost OR “economic burden”.

French keyword searches in PubMed used the following key words: Algérie OR algérien AND diabète OR diabétique OR neuropathie OR rétinopathie OR néphropathie OR “l'insuffisance rénale chronique” OR “pied diabétique” OR “mal perforant plantaire” AND “prevalence” OR “dépenses” OR “coût” OR “fardeau économique”.

In Web of Science, Scopus, Google Scholar and Google, varying combinations of the key words listed above were used in searches.

We did not put any time limits or language restrictions on our search. Articles that were retrieved through the literature review which did not present relevant evidence of the prevalence, costs, complications, treatment or outcomes of diabetes in Algeria were not retained for analysis. Studies that used both primary and secondary data were included. Where such information was available, we distinguished between type 1 and type 2 diabetes.

In addition to articles procured through the aforementioned search engines, relevant material known by the authors was consulted; this includes data from national, regional and local health surveys and diabetes studies. Data from the Algerian Ministry of Health, the International Diabetes Federation (IDF) and WHO were also consulted, along with government reports pertaining to national health policy and diabetes programmes. Finally, additional articles were identified from the references in the retrieved literature.

Given the limited reliable data available for diabetes and the absence of a data collection system at the national level, we attempted to fill in this gap by consulting experts, heads of specialist endocrinology-diabetology and internal medicine departments and directors of the Ministry of Health concerned with NCDs, as well as representatives of academic institutions and NGOs. Their input could overcome the shortcomings of non-aggregated data at the national level.

To update costs to 2013 United States dollars (USD), we used the Consumer Price Index (CPI) inflation calculator tool provided by the U.S. Department of Labor [8].

## Results

In PubMed, English keyword searches yielded 65 search results while French keyword searches did not yield any search results when using all of the keywords (some results were returned using various combinations of the keywords). In Web of Science and Scopus, English keyword searches yielded 23 search results and 46 search results, respectively, while French keyword searches yielded one search result and six search results, respectively. The majority of these papers were excluded through title screening and abstract screening because they did not meet inclusion criteria or were repeated results.

Ultimately, three papers were retained from PubMed, four papers from Web of Science and six papers from Scopus (Table 1). Through Google Scholar and Google, an additional three peer-reviewed papers were identified. Two more papers were identified through reference searching in other papers. The remaining references are comprised of grey literature, such as government reports and data from WHO and IDF, along with pertinent sources known by the authors.

**Table 1:** Literature

**Area of diabetes management**	**Number of references retrieved**	**References**
Prevalence	8	[9-12,16-19]
Costs	3	[14,15,26]
Complications	2	[13,21]
Treatment	2	[13,20]
Outcomes	1	[13]

### Data sources

A limited number of data sources on the burden, complications, costs and outcomes of diabetes in Algeria were identified. These include national and regional surveys, regional diabetes registers, a local register of renal replacement therapy and other studies.

#### National data

Major national studies that were identified include four studies on prevalence, two studies on costs and one study on complications, treatment and outcomes.

• National Health Survey: Conducted in 1990 by the National Institute of Public Health, the National Health Survey was a broad public health survey which involved a sample of 12,041 households covering all age groups. Diabetes patients were identified through self-reporting and population survey. There was no distinction between type 1 and type 2 diabetes [9].

• WHO STEPWise Approach Study: This study, using the “WHO STEPWise approach to surveillance” (STEPS), was conducted in 2003 under the auspices of the Ministry of Health and the direction of the National Diabetes Committee. It covered a sample of 4,050 adults aged 25 to 64 years in two pilot areas with the aims of assessing the frequency and distribution of risk factors for NCDs and the prevalence of diabetes, arterial hypertension cancer and chronic respiratory conditions [10].

• MICS 3 UNICEF: This study was conducted in 2006 on a sample of 29,008 women (aged 15 to 40 years) and children (under 5 years old) [11].

• TAHINA (Transition And Health Impact in North Africa) Study: Conducted in 2005 by the National Institute of Public Health, this national health survey used a representative sample of 4,818 adults aged 35 to 70 years. It is a comprehensive research project on Algeria’s epidemiological transition with the aims of estimating the level of morbidity (including prevalence and incidence), health care consumption and frequency of risk factors pertaining to chronic diseases among adults aged 35 to 70 years. Diabetes patients were identified through blood glucose testing. Only type 2 diabetes was studied [12].

• DiabCare Algeria: This is a prospective, multicentre transversal study on diabetes in Algeria that was funded by Novo Nordisk and conducted in 2008 by clinical researchers at six university hospital centres in the Central, East and West regions of the country. It assessed glycaemic control, risk factors, diabetes complications and therapeutic regimens based on an evaluation of 977 patients with both type 1 and type 2 diabetes [13].

• Priorités de santé en Algérie (Health Priorities in Algeria): Cost data in this 1998 study were collected from three university hospitals in Algiers; fixed costs (infrastructure, depreciation, personnel) were considered separately from variable costs (drugs, radiological examinations, laboratory tests). Information on resource utilisation was provided by a panel of physicians [14].

• Direct Medical Management Costs of Diabetes-Related Complications in Algeria: Costs in this 2012 study were collected from official sources identified using local resources. They were divided into six categories based mainly on type of complications (management costs, cardiovascular complications, renal complications, acute events, eye-disease and neuropathy/foot ulcers) and presented as first-year costs and costs in subsequent years following an event in 2011 USD [15].

#### Regional and local data

Four major regional studies and three regional registers on prevalence, in addition to one local register on treatment, were identified.

• Region “West” study: Conducted in 2007 in Tlemcen (Western Algeria) based on WHO criteria which used a sample of 7,656 subjects (2,799 men and 4,857 women) over 20 years of age [16].

• Region “East” survey: Conducted in 2001 in Sétif (eastern Algeria) on a sample of 1,457 subjects aged 30 to 64 years [17].

• Region “Centre” survey: Conducted in 2010 and involved 1,036 children under 15 years of age. By gender, the sample was comprised of 47.5% boys and 52.5% girls and the age category distribution was 12% (0–4 years), 34% (5–9 years) and 54% (10–14 years) [18].

• Region “South” (Sahara) survey: Conducted in 2002 of the “Touareg” population of the Algerian Sahara with a sample of inhabitants whose age and sex were not specified [19].

• Diabetes registers: There are three functioning and up-to-date regional registers for type 1 diabetes in children under 15 years of age. These registers are filled in by medical specialists, who also analyse and manage the data. The first register was created in Oran (Western region) in 1980, the second one was created in Constantine (Eastern region) in 2000 and the third register was created in Algiers (Central region) in 2010 – all by the National Institute of Public Health (which is also responsible for maintaining the registers). The Ministry of Health recently announced that record-keeping would be extended to 48 districts in the country and that a gestational diabetes register would be created. In addition to basic demographic information of the patient, these registers contain information on health indicators and treatment of diabetes (including incidence, mortality, date of diagnosis, quality of care and a range of tests for HBA_1c_, lipids, hypertension and weight), as well as complications arising from and/or linked with the disease. However, these registers do not contain data on the direct or indirect costs associated with diabetes (i.e. medicines, tests, etc.).

• Renal replacement therapy register: A register was created in 2009 in the city of Constantine and gradually improved over time [20]. The register collects basic patient information such as age, sex, blood group and place of care in addition to disability, co-morbidities and cause of death for all patients undergoing dialysis in Constantine.

#### Other studies

Additional smaller studies on complications [21]; risk factors [22,23]; consanguinity [24]; and fasting during Ramadan were identified [25].

### Prevalence and distribution of type 2 diabetes

While the national studies sampled different groups and regions and employed different methodologies, evidence from them suggests that the prevalence of diabetes in Algeria has increased from 6.8% in 1990 to 12.29% in 2005 (Table 2) [9,12]. In 2005, diabetes was the second most common chronic disease (8.78%) among 35-to-70-year-olds, preceded only by hypertension (16.23%) [12]. The national prevalence of diabetes in Algeria in 2013, according to the IDF, is estimated at 6.63% (the comparative prevalence is 7.47%) [26].

**Table 2:** Prevalence

**Prevalence of DM**					
**Estimate**	**Study year**	**Sampling frame and sample**	**Study design**	**Diagnostic test and diagnostic criteria**	**Reference**
DM2: 2.1% (1992), 8.7% (1994), 7.3% (2006)	2008	Multiple sampling frames and samples	Multiple study designs	Multiple diagnostic test and criteria	[3]
Overall: 6.8%	1990	Sampling frame: N/A	Cross-sectional	Diagnostic test: Self-reporting, population survey	[9]
		Sample: 12,041 households covering all age groups		Diagnostic criteria: N/A	
Overall: 8.9%	2003	Sampling frame: two pilot villages	Cross-sectional	Diagnostic test: Fasting blood glucose	[10]
		Sample: 4,050 adults aged 25 to 64 years		Diagnostic criteria: American Diabetes Association (ADA): The person is treated for diabetes or has a glycaemia level >1.26 g/l	
Overall: 2.1%	November-December 2005	Sampling frame: Four regions: Central, South, East, West	Cross-sectional	Diagnostic test: Self-reporting, population survey	[11]
Males: 1.9%					
Females: 2.3%					
Peak age group was 60+ (12.5%), followed by 35–59 (4.1%) and 25–34 (0.4%)		Sample: 25,919 females aged 15 to 49 years and 3,089 children under 5 years of age		Diagnostic criteria: N/A	
More prevalent in urban areas (2.6%) than in rural areas (1.5%)					
More prevalent among the richest quintile (3.5%) than among the poorest quintile (1%)					
Overall: 12.29%	June 2005	Sampling frame: Three regions: Tell, High	Cross-sectional	Diagnostic test: Glucose testing	[12]
Males: 11.93%					
Females: 12.54%		Plateaus, South (Sahara)		Diagnostic criteria: N/A	
Peak age group was 60–64 (22.37%), followed by 65–70 (22.05%) and 55–59 (13.70%)		Sample: 4,818 adults aged 35 to 70			
More prevalent in urban areas (13.81%) than in rural areas (9.62%)					
More prevalent in the High Plateaus (13.27%) than in the Tell (12.26%) or South (Sahara) (8.52%)					
Overall: 14.2%	2007	Sampling frame: Tlemcen (in western Algeria)	Cross-sectional	Diagnostic test: Fasting blood sample	[16]
Males: 20.4%					
Females: 10.7%		Sample: 7,656 adults (2,799 males, 4,857 females) over 20 years of age		Diagnostic criteria: WHO (1985)	
DM1: 3.7%					
DM2: 10.5%					
More prevalent in urban areas (15.3%) than in rural (12.9%) areas					
DM2: 8.2%	2001	Sampling frame: Setif (in eastern Algeria)	Cross-sectional	Diagnostic test: Oral glucose tolerance test	[17]
7.3% urban vs. 9.7% rural					
		Sample: 1, 457 subjects aged 30 to 64 years		Diagnostic criteria: WHO	
Overall prevalence: 13.8%	2010	Sampling frame: Central Algeria	Cross-sectional	Diagnostic test: N/A	[18]
		Sample: 1,036 children under 15 years of age (47.5% boys and 52.5% girls; age category distribution--12% (0–4 years), 34% (5–9 years) and 54% (10–14 years)		Diagnostic criteria: N/A	
DM2: 1.3%	2002	Sampling frame: Adrar (in southern Algeria)	Cross-sectional	Diagnostic test: N/A	[19]
		Sample: 1,000 subjects		Diagnostic criteria: N/A	

Similar observations regarding the prevalence and distribution of diabetes in the national studies were reflected in the regional studies. The 2007 Tlemcen study reported the prevalence of type 1 and type 2 diabetes at 14.2% in western Algeria [16]. The prevalence of type 2 diabetes (initially non-insulin-dependent diabetes mellitus) was 10.5% and type 1 diabetes (insulin-dependent diabetes mellitus) was 3.7% [16]. Diabetes prevalence was higher in urban (15.3%) than rural (12.9%) areas and higher among men (20.4%) than women (10.7%) [16]. The 2001 Setif study reported type 2 diabetes prevalence at 8.2%, increasing with age, but with no significant difference according to sex or urban (7.3%) / rural distribution in eastern Algeria (9.7%); half of the diabetes cases in this study had been previously undiagnosed [17].

The TAHINA study also found that the increase in morbidity as a result of NCDs such as diabetes, hypertension, chronic respiratory illnesses, chronic renal failure and cancer is associated with lifestyle changes in relation to food, obesity, physical activity and psychological stress. It was found that being overweight was a risk factor for type 2 diabetes and was observed in 55.9% of those surveyed. Furthermore, obesity was more common in women (66.5%), those aged 45 to 59 years (62.7%), those living in urban areas (58.8%) and those residing in the Central Highlands (57.8%) [12]. The Tlemcen study confirmed the link between obesity and diabetes prevalence, finding that 56.7% of all men and more than half of all women in urban areas were obese on the basis of body mass index (BMI) [16].

Two studies on risk factors in two areas in western Algeria further confirm the link between type 2 diabetes and changing lifestyle habits [22,23]. A 2007 study conducted in an urban community of Tlemcen observed significant prevalence rates of diabetes and obesity (16.1% and 19.2%, respectively) [22]. A study in Maghnia, conducted in 2008–2009, revealed that obesity, physical inactivity and irregular food intake were among the most important environmental risk factors associated with type 2 diabetes [23]. This study also noted that people with a family history of diabetes were at higher risk for developing the disease.

Additionally, the TAHINA study observed a low consumption of fruits, vegetables and fish (even in coastal areas) along with a high intake of processed foods, fats, sugars and grains. For physical activity, activities of low and moderate intensity were practised by 60.5% and 8.35%, respectively, of those surveyed [12].

In a 2009 case–control study of gestational diabetes, it was found that risk factors for the disease include family history of type 2 diabetes, being overweight and a history of polyhydramnios and/or macrosomia in previous pregnancies [27]. This study demonstrated a significantly higher number of Caesarean sections and premature births among pregnant women diagnosed with gestational diabetes compared to those without diabetes (type 1, type 2 or gestational). Gestational diabetes is associated with increased infant and maternal morbidity and mortality in the short term, and there is a significant risk of the mother developing diabetes (typically type 2) in the long term. Additionally, children born to mothers with gestational diabetes frequently are obese and have impaired glucose tolerance [27].

### Prevalence and incidence of type 1 diabetes in children

There are three regional registers collecting data on the incidence, prevalence and treatment of type 1 diabetes in children under 15 years of age. According to the data from Oran and Constantine, the incidence in 2000 was 12 cases per 100,000 people, representing approximately 1,146 new cases per year for the whole country based on census figures from 2008 [28].

The register for Algiers (Central region) reported the prevalence of type 1 diabetes to be 13% in 2010. When broken down by age group, type 1 diabetes prevalence is: 4.4% of children under 5 years of age, 15.4% of 5–9-year-olds and 23.5% of 10–14-year-olds. The incidence in 2011 was 22.8 cases per 100,000 people, representing 171 new cases for the Central region alone and increasing among younger people, including infants. Family history is a contributing factor in half of these cases.

### Diabetes complications

While there are limited available data and no baseline studies on diabetes complications, we were able to identify two relevant studies [13,21] (Table 3). Additionally, we consulted with an array of Algerian public health officials and medical specialists.

**Table 3:** Complications

**Estimate**	**Study year**	**Sample frame and sample**	**Study design**	**Diagnostic test and diagnostic criteria**	**Reference**
**Diabetic retinopathy**	2008	Sample frame: Three	Clinical screening	Diagnostic test: Blood samples	[13]
34.5% (N = 977, n = 300)		regions: Central, East, West		Diagnostic Criteria: ADA	
DM1: 36.2% (n = 46)		Sample: 977 diabetes patients with DM1 (n = 136) and DM2 (n = 841)			
DM2: 34.2% (n = 254)					
**Diabetic neuropathy**					
44.7% (N = 977, n = 433)					
DM1: 35.8% (n = 48)					
DM2: 46.2% (n = 385)					
**End-stage renal disease**					
.5% (N = 977, n = 5)					
DM1: 0% (n = 0)					
DM2: .6% (n = 5)					
**Diabetic retinopathy**	Dec 2008-Jun 2009	Sample frame: Algiers province	Cross-sectional	Diagnostic test: N/A	[21]
DMT1/2: 48.6% ± 2.9%					
				Diagnostic Criteria: Alfediam criteria (Association de langue française pour l'étude du diabète et des maladies métaboliques)	
Males: 49.6%					
Females: 50.4%		Sample:			
		1,152 diabetes patients; 15 years of age or older with DM1, DM2 insulin-treated (IT) and DM2 non-insulin-treated (NIT) diabetes. Divided into three groups (n = 384) based on disease duration (less than 5 years, between 5–10 years, longer than 10 years).			
DM1: 47.9% ± 7.0%					
DM2 (IT and NIT.): 48.7% ± 5.2%					
DMT2 (IT): 70.6% ±4.6%					
DMT2 (NIT): 35.2% ± 3.8%					
Proliferative diabetic retinopathy: 10.9% ± 1.8%					

The 2008 DiabCare Algeria study was conducted on 977 patients with both type 1 and type 2 diabetes. The majority of patients (86.1%) had type 2 diabetes, were young (mean age: 48.5 years), overweight (mean BMI: 27.7 kg/m2) and with a disease duration of 10 years. The 2009 diabetic retinopathy study was conducted on 1,152 diabetes patients (type 1 and type 2 diabetes) 15 years of age or older in Algiers province [13].

According to the DiabCare study, the most common complication was diabetic neuropathy (diagnosed in over 44% of patients), followed by diabetic retinopathy (a third of patients). One in five patients had a micro-albuminuria and a little over a third of patients had macroproteinuria. End-stage renal disease was found in 0.52% of type 2 diabetes patients. Stroke was found to be more frequent in type 2 diabetes (2.6% vs 1.5% in type 1 diabetes) [13].

The diabetic retinopathy study found that this complication had a prevalence of 48.6% (type 1 and type 2 diabetes) and that it increased with disease duration [21]. According to expert opinion, monitoring of eye problems related to diabetes is very limited due to a lack of adequate ophthalmic services.

In consulting with NCD specialists at the Ministry of Health, we found that diabetic neuropathy and damage to the lower limbs occurs in 50% of people with diabetes. Additionally, 25% of cases of patients with arthritis of the lower limbs have been found to be linked to diabetes, with a quarter of such cases requiring amputation; the number of these amputations per year was estimated to be between 800 and 1,300.

According to cardiologists who were consulted, as well as past Algerian newspaper articles published on World Diabetes Day, it is estimated that 50% of diabetes patients have cardiovascular complications. These occur two to three times more often and with greater severity in people with type 2 diabetes when compared to people without the disease.

Consultations with nephrologists revealed that 14% of kidney failure is due to diabetes and that in 2011, approximately 2,000 people with diabetes had to undergo dialysis (this figure was relatively low due to the fact that only around 5% of the population is 65 and over [4]). The incidence of renal failure is approximately 50 to 100 new cases per year. Given the current growth rate of diabetes, the projection of new cases is expected to increase.

### Costs of diabetes and complications

There are limited reliable data on the costs of diabetes and its complications, and the currently available data are often fragmented or combined with other types of medical costs. As a result, only two relevant studies were identified in addition to the 2013 estimates developed by the IDF (Table 4).

**Table 4:** Costs

Source of cost*	**Original amount (USD) [Year]**	2013 US Dollar (USD) amount**	**Reference**
Average treatment costs per year per patient	351 [1998]	503	[14]
Average total treatment costs per year	11.6 million [1998]	16.6 million	
Renal transplantation	28,422 [2010]	30,441	[15]
Continuous ambulatory peritoneal dialysis	3,901 [2010]	4,178	
Haemodialysis	3,742 [2010]	4,008	
Myocardial infraction	865 [2010]	926	
Peripheral vascular disease	132 [2010]	141	
Stroke	282 [2010]	302	
Congestive heart failure	244 [2010]	261	
Angina	395 [2010]	423	
Amputation procedure	533 [2010]	571	
Prosthesis	618 [2010]	662	
Follow-up post- amputation procedure	22 [2010]	24	
Cataract operation	123 [2010]	132	
Laser eye procedure	48 [2010]	51	
Health expenditure for diabetes in 2013	512,682,393***	--	[26]
Health expenditure for diabetes in 2035	813,889,950***	--	

*First-year treatment costs unless otherwise indicated.

**Costs updated to 2013 USD using U.S. Department of Labor’s Consumer Price Index (CPI) inflation calculator tool: http://www.bls.gov/data/inflation_calculator.htm.

***Authors’ calculation based on IDF data.

The 1998 study was conducted by local researchers using the methodology of the World Bank and incorporating the disability adjusted life years concept [14]. Cost data in this study were primarily collected from three university hospitals in Algiers. This study determined that the treatment of type 1 diabetes in Algeria cost on average USD 351 per year (60% of this was on drug expenditure). Based on an estimated prevalence of 7.76% in the age affected group (which amounted to 33,184 cases), the total cost came to USD 11.6 million.

The 2010 study observed that the highest first-year costs were for renal complication treatments: renal transplantation (USD 28,422), continuous ambulatory peritoneal dialysis (USD 3,901) and haemodialysis (USD 3,742) [15]. Cardiovascular complications also incurred high annual costs and ranged from USD 865 for first-year treatment of myocardial infarction to USD 132 for first-year treatment of peripheral vascular disease. Additional first-year costs of treating cardiovascular complications were identified for stroke (USD 282), congestive heart failure (USD 244) and angina (USD 395). Also, the cost of an amputation procedure was USD 533, excluding the cost of prosthesis (USD 618), with a follow-up cost of USD 22. The cost of a cataract operation was USD 123, while the cost of a laser eye procedure was USD 48.

The methodology used by the IDF to derive estimates was based on information on country-by-country estimates of diabetes prevalence by age and sex, population size by age and sex, total healthcare expenditures by age and sex and the ratio of expenditures per person with diabetes to expenditures per person without diabetes, matched for age and sex [29].

According to the IDF, spending on diabetes per patient was USD 313 in 2013, representing a total spending of nearly USD 513 million for the Algerian health care system [26].

### Screening and prevention of diabetes

Some screening has been implemented systematically in all diabetes treatment centres such as day hospitals, but it is less frequent in polyclinics. Screening is targeted to high-risk groups, defined as patients age 35 and older with a family history of the disease, obese patients and women who have delivered babies with a high birth weight. Regarding gestational diabetes, screening is not currently performed as part of standard antenatal care, so early detection is absent both for women in general and women at risk [27].

The Ministry of Health has developed disease prevention programmes based on WHO’s integrated approach; these have focused on the identification and monitoring of diabetes risk factors.

There are also many diabetes associations at the national and regional levels which play an active role in disease prevention. Scientific societies such as the Algerian Society of Diabetology continue to raise awareness about the importance of screening as well as healthy lifestyles, improved diets and increased activity levels. In addition to that, pharmaceutical companies are also involved in diabetes screening and prevention activities.

### Treatment

In treating diabetes, the medical community abides by international standards established by WHO. Additionally, the guide to best practices for diabetes prevention that was developed by the National Diabetes Committee has served as a reference tool.

During the 12 months preceding the 2008 DiabCare study, 18% of patients had received education on treatment regimens and only 56.6% of type 1 and 63.1% of type 2 diabetes patients benefited from the use of dietary guidelines [13]. HBA_1c_ test was performed on average three times per year. Therapeutically, 80.1% of type 2 diabetes patients were on an oral anti-diabetic (OAD), 26.3% were on OAD and insulin and 19.6% were on insulin only. Biguanides were the OAD class most frequently prescribed (63.5%), followed by sulfonylureas (44.0%), meglitinides (glinides) (6.7%) and α-glucosidase inhibitors (1.4%). The type 2 diabetes patients received an average insulin dose of 42.2 ± 25.2 U/day (0.6 ± 0.3 units/kg/d) with an average number of injections of 2.0 ± 0.9 U/j (58.1% of them used insulin pens). The type 1 diabetes patients received an average insulin dose of 58.2 ± 22.5 U/day (0.9 ± 0.3 units/kg/d) with average of three injections per day (half of them used insulin pens).

Patients with diabetes are treated and rehabilitated in public hospitals that specialise in internal medicine and endocrinology, public diabetes treatment centres and private specialist clinics and medical offices. Because public health care is free, most patients with diabetes receive their treatment from public hospitals. To have routine medical tests performed, patients can visit public hospitals as well as private medical testing laboratories; expenses are reimbursed by social insurance. Lower limb prostheses for insured patients are covered by social security under specialised services, while non-insured patients must pay directly for this equipment (however, national NGOs generally cover these costs for them). Dialysis is available in both public and private centres (the former is free-of-charge, the latter is paid for by social security and/or health insurance).

There are approximately 20 specialised hospitals that treat only diabetes in Algeria, playing an important role in the prevention and treatment of this disease. In these facilities, diabetes patients have access to high-quality, multidisciplinary therapeutic treatment, including consultations, investigations and screening of complications, provided by endocrinologists, diabetologists and internists. Additional services include: dietary advice to promote eating healthy and nutritionally balanced meals; training in the use of insulin and the glucose monitor; psychological support; eye and cardiologic exams and diabetic foot care; and social assistance for obtaining medicines. Diabetes patients are regularly monitored by their doctor on an average bi-monthly basis.

The Algerian drug market is adequately supplied with insulin, oral medications and test strips. A significant portion of insulin has been locally produced and will be further developed through local investment. Many oral medications such as novoformine are produced locally in generic form, and imports supplement domestic production.

Access to medicines is generally considered satisfactory. The insured and their beneficiaries, along with welfare recipients in possession of social cards, obtain their medications free-of-charge in pharmacies that have established agreements with the two main health insurance organisations. Additionally, all chronically ill patients employed in the formal sector, including people with diabetes, have an electronic card that allows them to obtain prescribed drugs free-of-charge in pharmacies. Test strips are procured in the same manner as prescription drugs and patients are reimbursed for 100% of the cost of test strips; the price is fixed by their doctor according to the daily number of test strips, added up to the monthly number of test strip boxes that a patient is expected to use. Glucose meters, however, are not reimbursed (though NGOs provide these at no cost to those who cannot afford them). Additionally, patients are reimbursed for 100% of the cost of insulin in all its forms (injection pens, pumps, etc.).

According to a local register of renal replacement therapy from the city of Constantine, conventional haemodialysis is the most frequently used method for renal replacement therapy, comprising 90% of cases (this figure includes diabetes as well as non-diabetes patients) [20]. Renal transplantation, as a form of renal replacement therapy, does not appear to be widespread, largely because of the unacceptability of cadaveric donors (due to ethical questions raised by Islamic law) which limits organ supply to living donors.

### Health outcomes

Data on health outcomes for diabetes patients are limited, but a few sources provide some insight into disease control and mortality.

In the DiabCare Algeria study (2008), glycaemic control was poor with an average HbA_1c_ of 8.52%, and more than one third of the patients studied had a HbA_1c_ level higher than 9% [13]. Only 18.7% of diabetes patients reached HbA_1c_ <7%.

Diabetes also contributes to mortality. In Algeria in 2010, diabetes accounted for 4% of all deaths [1]. According to the IDF, 14,431 deaths have been attributed to diabetes in 2013 [26].

### Consanguinity and fasting

When examining a particular disease in a specific country context, it is important to also consider cultural issues which may affect the burden and management of the disease. Regarding diabetes in Algeria, religious fasting (during Ramadan) and consanguineous marriages are two important issues to consider.

Algeria is 99% Muslim, and as in all majority-Muslim countries, the holy month of Ramadan is one of the most important holidays for the country’s inhabitants [4]. From dawn to sunset during Ramadan, followers of Islam fast by entirely abstaining from eating, drinking water (or any other liquid), taking oral medications and smoking. Once they break the fast at sunset, people consume one to three meals, with some people snacking until dawn; the foods consumed between sunset and dawn tend to be high in sugar and fat. While Ramadan allows exemptions from fasting for high-risk and ill individuals, including people with diabetes, the majority of them still insist on fasting [25]. During the holy month, in fact, severe hypoglycaemia is the main cause of hospitalisations. Diabetes patients in Algeria face additional complications such as ketoacidosis, dehydration, orthostatic hypotension and thrombosis when fasting for Ramadan [25].

The practice of consanguineous marriages has not been extensively studied in relation to the prevalence of diabetes, despite the fact that in Algeria (as in many other North African as well as Middle Eastern countries), consanguineous marriages (unions between closely-related individuals) are not uncommon. According to a 2007 Algerian study by the National Foundation for Health Promotion and Research Development, one Algerian in four is married to a cousin [30]. To establish the profile of people at high risk of type 2 diabetes, a study of 1,561 subjects was conducted in 2010 across five regions and cities in Algeria using a logistic model [24]. In the overall sample, the rate of consanguinity was found to be 48%. This study concluded that consanguineous marriages, along with hereditary predisposition and obesity, were significant risk factors for the disease [24].

### Health policy and diabetes programmes

The government of Algeria is concerned about the concomitant changes that have resulted from the growth of NCDs and the increased social demand for quality care.

In 2003, the Minister of Health at the time emphasised that only primary and secondary prevention through education and the promotion of healthy lifestyles, screening and early treatment of cases offer effective and relatively inexpensive means of significantly reducing the burden of NCDs [31].

National health policy, in an effort to combat the growing diabetes epidemic, has aimed to re-direct resources towards correcting regional imbalances in the supply of care (i.e. hospital infrastructure and health personnel) and the management of NCDs by building specialised hospitals and by training specialists and skilled workers. It also aims to preserve the free care provided by the government for low-income individuals and to streamline the entire health care system.

The draft national plan against diabetes for 2011–2020 was adopted by Algeria’s Parliament in 2012, but the legislative presidential decrees have not yet been developed and signed. This plan is mainly characterised by the fact that it is integrated into the global WHO approach to NCDs (which is general and based on several global studies) that is founded on prevention at the primary (targeting risk factors), secondary (screening) and tertiary (prevention of complications) levels. The plan differs from the previous strategy in its multidisciplinary action that incorporates a number of ministerial departments and emphasises awareness, training, prevention and integrated control against risk factors in order to reverse the upward trend of the disease. The allocation of a special budget solely to the fight against diabetes is expected to help address regional inequalities in terms of providing and ensuring equity of care for patients.

Policies addressing the rise in obesity that is a major contributing factor to the increased prevalence of type 2 diabetes appear to be limited. There are occasional televised advertisements that call attention to the negative health effects of cheap, processed food. However, taxes are not levied on such “junk” food and there is no regulation on food advertising to children.

## Discussion

Our study was limited by the lack of reliable and extensive data. There do not appear to be many recent national or regional studies on diabetes mellitus in Algeria that have been published or made publicly available. This may be partially attributed to the absence of an integrated information system and the focus, until recently, on infectious diseases (though the public health context is changing). Additionally, the databases from which we collected various statistics and costs cite the methodological challenge of basing their estimates on insufficient national data; this is especially pertinent to developing countries such as Algeria.

This study has found that the epidemiology of diabetes is continually evolving. According to forecasts of the Ministry of Health, there were 4.4 million people with diabetes in Algeria in 2012, and this estimate is projected to increase to 4.8 million by the year 2015. (The Ministry of Health’s estimates are based primarily on numbers of known diabetes patients, but the numbers of those undiagnosed would certainly increase these figures).

Diabetes now affects all population groups in Algeria, with an estimated national prevalence rate for those ages 20 to 79 of 6.63%, resulting in a 1.7 million patients [26]. This age group constitutes a large segment of the working population whose households could be severely affected economically, to the point of patients being forced to make difficult choices between basic necessities (food, shelter, etc.) and health care.

Although 90% of the Algerian population is covered by a social insurance scheme, there are nearly one million workers (such as artisans) in the informal sector whose income goes unreported and untaxed and who, in turn, do not receive government health benefits [7]. When additional family members and their dependents are taken into account, the number of those without any coverage reaches approximately 3.5 million, according to the Algerian Ministry of Labour, Employment and Social Security. Based on an approximate diabetes prevalence of 10% (as an average of studies discussed earlier), it is estimated that approximately 350,000 patients are not specifically covered for payment of diabetes treatment. The estimated one million people working in the informal sector earn an income and procure their medication and care from the private sector; these are non-reimbursed expenses, which patients must pay for out of pocket.

The geographical features of Algeria reflect the organisational and demographic challenges that are currently facing health care providers and planners, particularly regarding the risk of chronic disease. Regional disparities appear in the context of specialised care. There is a general shortage of endocrinologists, diabetologists and internists across regions. The Ministry of Health has sought to fill this gap by training general practitioners in poorer areas to enable them to treat and educate patients diagnosed with diabetes. Because there are not enough diabetologists, the Ministry of Health provides additional training for GPs working in remote areas to treat diabetes. The training of general practitioners is an ongoing mission to first detect diabetes (particularly type 2) in people, and then to dispense advice on diet and exercise to those who are diagnosed; more intense treatment regimens are provided in specialised hospitals.

Deficiencies found in the care of diabetes patients are most acute when there are complications. Hospital departments providing specialised treatments (i.e. foot care, cardiology, nephrology, ophthalmology) are not always able to accommodate these additional patients. Additionally, despite broadening efforts, screening policy is marred by deficiencies in its organisation, standardisation and communication. There are no routine screening procedures in place in doctors’ offices or private consulting services for major hospitals.

Diabetes poses a significant burden on the economy. According to forecasts of the Algerian Ministry of Health, national health expenditure has more than doubled since 2001. The amount spent on diabetes is expected to rise to as high as 816 million by 2035 [15]. Costs related to treatment for complications of diabetes are expected to add to the financial burden of social insurance programmes in Algeria. Since type 2 diabetes affects a significant proportion of the population aged 35 to 60 years, and people are now living longer than they did a few generations ago (life expectancy at birth is 71 and 74 years of age for males and females, respectively), there clearly are negative implications for limited public resources, particularly in regard to social security [1].

Absence from work due to illness (absenteeism) and premature death considerably reduce the national income, while early retirement for workers and sick pensions for invalids further compound the burden on financial resources allocated to social security. Furthermore, households can be doubly burdened because the disease increases the risk of job losses and care-related expenses, which can prove to be unbearably costly for low income or informal sector workers.

This predicament is further exacerbated in an emerging country like Algeria, which is more likely to face serious financial instability that can potentially interfere with economic growth and development efforts.

## Conclusion

According to the available published literature and majority of the diabetologists consulted, the shift from the traditional life style to a more Westernised one is the major explanatory factor behind the rapid progression of diabetes mellitus, particularly type 2, in Algeria. Indeed, residents in urban and even rural areas have become more sedentary, exercise less and have replaced their consumption of foods rich in fibre, such as vegetables and fruits, with cheaply-priced products that are readily available in the market place and are high in saturated fats and refined sugar. This has led to a significant increase in obesity.

While access to health care services, at least for insured patients, is fairly good, health outcomes appear to be quite poor. Screening and prevention initiatives are expanding, but they remain poorly organised and poorly coordinated; given that these represent the most effective approach to reducing the complications of the disease and its serious socio-economic consequences, this must be addressed.

Since diabetes is now a widespread public health concern, the development of an integrated control strategy that would help reduce its morbidity and mortality rates and maintain cost control is essential. When considering prevention and treatment strategies for chronic diseases such as diabetes, Algerian health policymakers should prioritise health system changes that are structural (reorganisation of health care services and specialist training), operational (review of action plan strategies) and financial (budgetary allocation).

To tackle the obesity problem, policymakers, public health experts and medical professionals should promote the consumption of a balanced, nutritious diet as well as a regular exercise regimen. It should be noted, however, that advocating healthier eating might pose financial obstacles for some households that can only afford the cheaper, less healthy food options. Despite the gradual improvement of general living standards, some low-income households affected by diabetes have difficulty maintaining a diet that regularly includes pricier foods.

Key policy recommendations are:

1. Train GPs across the country to take a more active role in the screening of high-risk groups, treatment and management of type 2 diabetes. This should help decrease late diagnosis and the frequency of patients seeking care at higher (and costlier) levels when such care can instead be delivered at the primary level.

2. Gradually move towards integrated care of NCDs and increase efforts to improve screening for complications.

3. Strengthen and integrate existing data collection systems to allow monitoring of risk-factors, morbidity and mortality. Ultimately, this will also enable the evaluation of health programmes and reforms to tackle not only NCDs but also other conditions.

4. Allocate adequate public resources and devise government programmes that specifically target diabetes (particularly type 2). This includes media campaigns promoting healthier living, including proper diet and exercise.

## Abbreviations

ADA: American Diabetes Association; BMI: Body mass index; CNAS: Caisse Nationale des Assurances Sociales des Travailleurs Salariés; CPI: Consumer price index; IDF: International Diabetes Federation; IT: Insulin-treated; NCD: Non-communicable disease; NIT: Non-insulin-treated; OAD: Oral anti-diabetic; TAHINA: Transition and health impact in North Africa; USD: United States Dollars; WHO: World Health Organization.

## Competing interests

AF received travel reimbursement and speaker fees from Novo Nordisk for delivering two presentations on diabetes in EU5 at national diabetes conferences in Portugal and Spain. The authors declare no conflict of interest.

## Authors’ contributions

All authors contributed to the literature review and in the appraisal of the retrieved information. LL conducted interviews with local experts. LL wrote the first draft of the article, EG redrafted subsequent versions with input from AF and LL up to its final version. All authors approved the final manuscript.
